# Designs and Algorithms to Map Eye Tracking Data with Dynamic Multielement Moving Objects

**DOI:** 10.1155/2016/9354760

**Published:** 2016-09-20

**Authors:** Ziho Kang, Saptarshi Mandal, Jerry Crutchfield, Angel Millan, Sarah N. McClung

**Affiliations:** ^1^School of Industrial and Systems Engineering, University of Oklahoma, 202 West Boyd Street, Norman, OK 73019, USA; ^2^Aerospace Human Factors Research Division, Civil Aerospace Medical Institute AAM-520, Federal Aviation Administration, P.O. Box 25082, Oklahoma City, OK 73125, USA; ^3^School of Electrical and Computer Engineering, University of Oklahoma, 110 W. Boyd Street, Devon Energy Hall 150, Norman, OK 73019-1102, USA

## Abstract

Design concepts and algorithms were developed to address the eye tracking analysis issues that arise when (1) participants interrogate dynamic multielement objects that can overlap on the display and (2) visual angle error of the eye trackers is incapable of providing exact eye fixation coordinates. These issues were addressed by (1) developing dynamic areas of interests (AOIs) in the form of either convex or rectangular shapes to represent the moving and shape-changing multielement objects, (2) introducing the concept of AOI gap tolerance (AGT) that controls the size of the AOIs to address the overlapping and visual angle error issues, and (3) finding a near optimal AGT value. The approach was tested in the context of air traffic control (ATC) operations where air traffic controller specialists (ATCSs) interrogated multiple moving aircraft on a radar display to detect and control the aircraft for the purpose of maintaining safe and expeditious air transportation. In addition, we show how eye tracking analysis results can differ based on how we define dynamic AOIs to determine eye fixations on moving objects. The results serve as a framework to more accurately analyze eye tracking data and to better support the analysis of human performance.

## 1. Introduction

Eye tracking research is useful for evaluating usability or analyzing human performance and more importantly understanding underlying cognitive processes based on the eye-mind hypothesis [1]. This hypothesis asserts that what we observe when performing a task is highly correlated with our cognitive processes. Thus, eye tracking research has been conducted in diverse fields to investigate how objects or spatially fixed areas are interrogated [2–7]. For example, an air traffic control specialist (ATCS) must timely detect and control multiple aircraft on a radar display in order to maintain a safe and expeditious flow of air traffic. Through eye tracking data, we can identify which aircraft the ATCS interrogates and what visual search pattern the ATCS applies.

However, the analysis of eye tracking data for a task that requires interrogating moving objects (e.g., an ATCS controlling multiple moving aircraft on a radar display or a weather forecaster determining whether to issue a warning by observing the weather features on a radar display) can be difficult due to the different characteristics of the moving objects and the limited capabilities of the eye tracking system. Furthermore, eye tracking analysis becomes more difficult if the object's overall shape can change due to the shape change of the object's elements or the physical relocation of its elements (e.g., an aircraft on a radar screen is composed of elements such as a vector line and a data block, and the length of the vector line can change due to the aircraft speed change, or the data block can be repositioned by the ATCS). The details of the issues are as follows.

**Figure 1:**
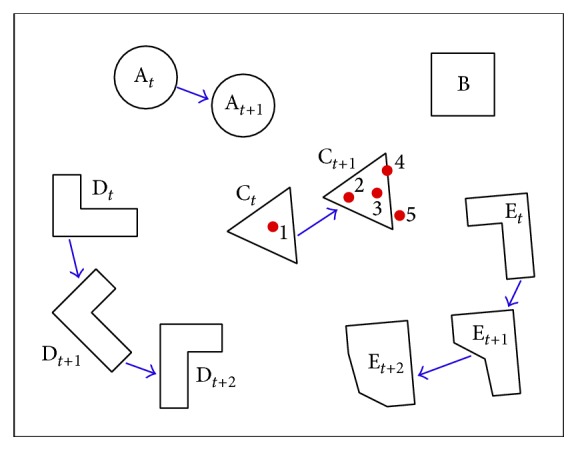
Characteristics of multiple moving objects: each object is in motion except for object “B.” “A_*t*_” indicates a circular object at time *t* and “A_*t*+1_” indicates the change of its location at time *t* + 1. “D” is an object rotating clockwise, and “E” is an object changing its shape. The red dots on and around “C” indicate the order of eye fixations at times *t* (eye fixation 1) and *t* + 1 (eye fixations 2 to 5).

In order to map and analyze the eye tracking data for such a task described above, different characteristics of those moving objects need to be identified (Figure 1). Objects can have irregular shapes and sizes and different movement characteristics and can be at close proximity or overlap with one another as time progresses. When the eye fixation data is collected, we can overlay the data with the objects to determine whether an eye fixation occurred on the object.

Eye tracking systems return pixel-based coordinates where the eyes fixated; however, we are more interested in (1) whether eye fixations occurred on the objects of interests as well as (2) the order of the eye fixations among those objects of interest. Specifically, we need to consider the following issues when mapping the pixel-based eye fixations with the multielement objects on a display.

**Figure 2:**
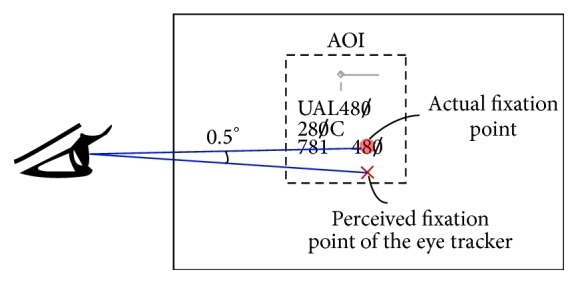
Area of interest (AOI) and visual angle accuracy error: The AOI approximates the shape of the object and should be slightly bigger than the original object size considering the visual angle error. The object consists of the aircraft itself (shown as a small diamond shape), the direction indicator (currently flying east), the data block (aircraft ID: UAL 480, altitude: cruising at 28000 ft., computer ID: 781, and speed: 480 knots), and the leader line which points to its corresponding aircraft.

One of the difficulties with mapping the eye tracking data to the objects is due to the visual angle accuracy of the eye trackers (Figure 2). A visual angle accuracy (expressed in degrees) is defined as the deviation of coordinates, collected from the eye tracker, from the actual location on which the individual fixated [8, 9] (e.g., 0.4~0.5° [10–12]) when using displays that are approximately below 16 (horizontal length) × 12 (vertical length) inches (or 22 inches diagonally) in size. For example, if a display is observed from 1 meter away with visual angle accuracy of 0.5°, then we can have up to 1 cm of error where the eyes fixated on. Therefore, observing the eye fixations shown as red dots in Figure 1, in addition to the first four eye fixations, we could also determine that the fifth eye fixation may have occurred on object “C.” In addition to the inherent error of eye tracking systems, accuracy error can also be affected by experimental conditions.

For example, in the actual air traffic control rooms, ATCSs sit close to a large monitor (i.e., 19.83 × 19.83 inches) in order to better detect and control multiple (i.e., sometimes up to 50 or more) aircraft within their sector. For such an environment, the accuracy of the eye tracker can drastically decrease. These issues occur when measuring eye tracking data not only in an air traffic control task, but also in other various tasks such as during driving or during a virtual simulation of offshore oil and gas operations. Therefore, the visual angle accuracy is not fixed at 0.5° and can vary based on the experimental conditions when we pursue high face validity.

In addition, the mapping of eye tracking data to moving objects can be difficult if there are multiple small objects moving on the display and each object is composed of several elements (e.g., the aircraft position symbol (or target), vector line, and data block). To accommodate the complex shapes of objects as well as the visual angle accuracy, the concept of an area of interest (AOI) can be applied. An AOI is a convex shape that can approximate and represent the complex object shape and can be simple shapes such as circles and rectangles. For example, the AOI can be fixed rectangular areas [5, 6, 13] or moving rectangular areas [9, 14] on a display based on the task types. Note that the size of an AOI should be slightly enlarged to consider the visual angle accuracy [9, 14].

**Figure 3:**
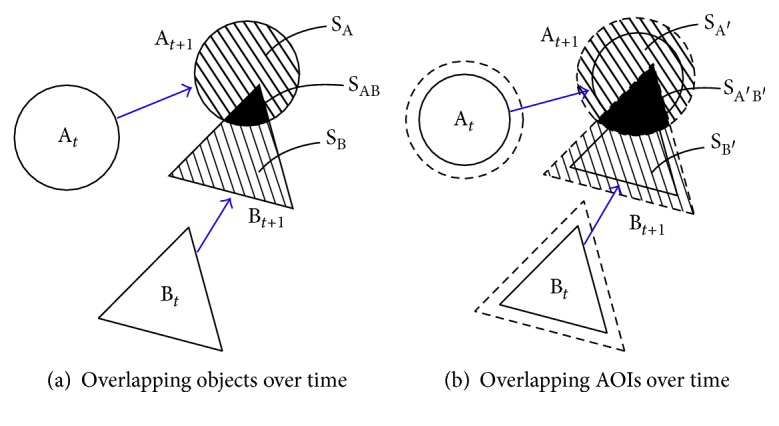
Overlapping objects and defined AOIs over time: the AOIs are designed slightly larger than the objects themselves to accommodate the visual angle error. The overlapping areas are denoted as S_AB_ and S_A′B′_.

To determine whether an eye fixation occurred on an object, we need to consider two aspects. First, the eye fixation should have occurred within the visual angle error range (e.g., 0.5° from all edge points of an object). Second, there should be no other object or background image to which the eye fixation occurred. In other words, if two objects are in close proximity, it can be difficult to determine which object the participant was interrogating. Even if the objects arrived from different locations, they can come into close proximity and even overlap as time progresses (Figure 3). Although considerable research was conducted to investigate the eye movements of air traffic control operations [15–18], it was limited to creating spatially fixed AOIs or did not elaborate on how overlapping issues were addressed.

**Figure 4:**
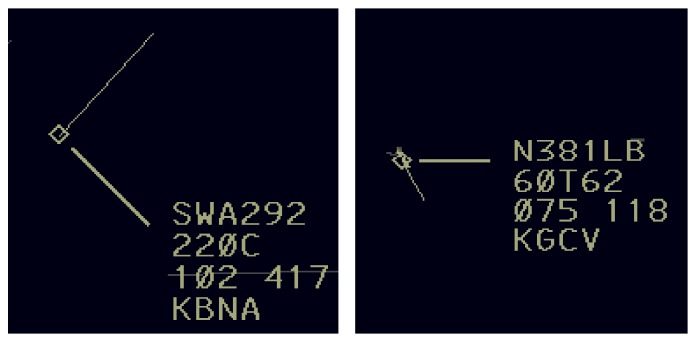
The overall shape change of an aircraft position indicator along with the data block. An ATCS can freely move around the data block since the leader line connects the data block to its aircraft. The overall shape also changes if the aircraft changes its direction.

Additionally, the mapping issue becomes more complex if the shapes of the multielement objects change. For example, if two aircraft are approaching close proximity, the aircraft position symbols (or targets) as well as the data blocks can overlap, and then an ATCS can reposition the data block (Figure 4). The data block can be repositioned in eight directions relative to the aircraft position symbol (e.g., from the bottom of the target itself to the top or right upper corner of the target) as well as increased in distance (e.g., from 0.5 cm away to 5 cm away).

In this paper, we present designs and algorithms to address the issues raised to facilitate the analysis of the eye tracking data for tasks that involve interrogating multielement moving objects that can change their overall shape and overlap with one another by considering different shapes and sizes of the AOIs that are fitted to represent the objects.

## 2. Conceptual Designs and Algorithms

**Figure 5:**
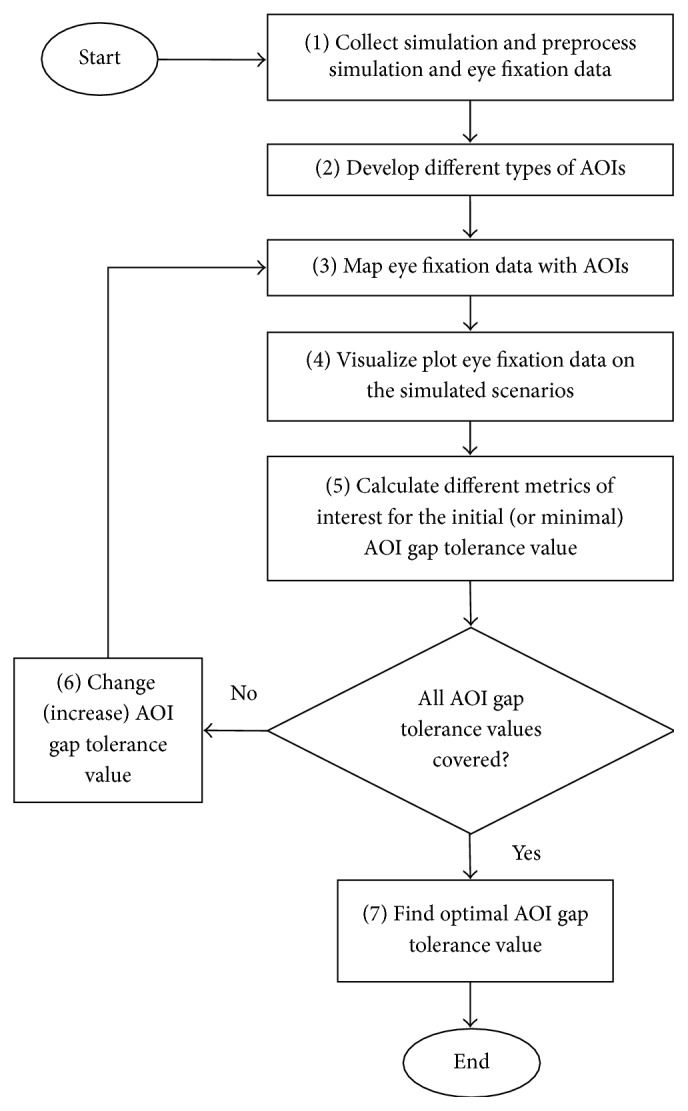
Data processing flowchart.

The main features of our approach are to (1) develop dynamic AOIs that continuously fit the multielement objects into convex or rectangular shapes whenever the objects' overall shapes or locations change, (2) modify the size of the AOIs (through the concept of AOI gap tolerance) to consider the visual angle error, (3) map the pixel-coordinate based eye fixations with the AOIs, and (4) define eye fixations on overlapping AOIs. Specific to air traffic control operations, the designs and algorithms create AOIs based on matching the pixel-coordinates from the flight data block, target, and vector lines with the pixel-coordinates of the eye fixations.  Figure 5 represents the data processing flowchart of the overall methodology. The flowchart consists of seven major steps, which are discussed in detail in the subsequent sections. Note that the introduced algorithm is based on discretized movements of the moving objects, and the background (scene) is fixed.

**Step 1 .**

Collect and preprocess simulation and eye tracking data.

*Step  1.1 (collect and preprocess simulation data).* Assume the simulation scenario is of *m* duration in minutes, given an update rate (UR) in seconds (e.g., 1 second), defined as the refresh rate of the objects' locations and shapes on a display; the total duration of a scenario can be divided into UR × *m* × 60 time frames in seconds. Thus, if we want to represent the *m* minutes scenario into discrete time frames we can represent it as

(1) $$\begin{array}{c} T=\left\{\;UR,UR\times 2,UR\times 3,\ldots ,UR\times m\times 60\;\right\}, \end{array}$$

where *T* represents the time frame counter in seconds.

**Figure 6:**
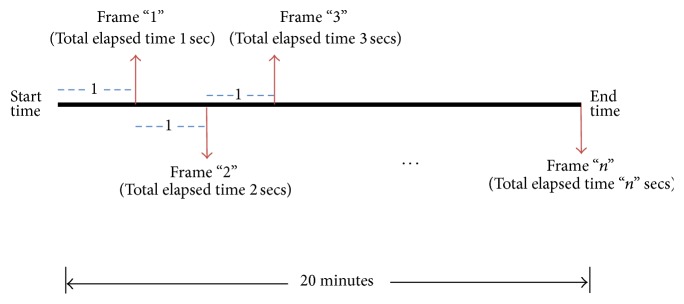
Discretization of the simulation video into time frames.

Figure 6 represents an example of the discretization process of the simulation output for a 20-minute duration. Note an observable (or systematic) discrete movement of the object (e.g., aircraft or radar display). In other words, no change in position occurs within a time frame; for example, suppose the simulation starts at 0 seconds, the next change in positon of the aircraft will occur at the end of the first second, and the next change will be at the end of two seconds and so on. After discretizing the time frames as part of the simulation data preprocessing step, the corresponding multielement object data are identified for each time frame. Let *P* be the set that contains all the information of the multielements for the total time duration. Then *P* can be represented as

(2) $$\begin{array}{c} P_{N_{T},T}=\left\{\;p_{n_{UR},UR},p_{n_{UR},UR\times 2},\ldots ,p_{n_{UR\times m\times 60},UR\times m\times 60}\;\right\}, \end{array}$$

where *P*_*N*_*T*_,*T*_ is the set of multielement objects present for each time frame.

*Step  1.2 (collect and preprocess eye fixation data).* The eye fixation data needs to be processed according to the time discretization strategy used for processing the simulation data.  Table 1 represents a small sample of eye fixation data. The first and second columns represent the horizontal and vertical pixel-coordinates of the eye fixations, respectively. The third and fourth columns show the start and stop time of an eye fixation. The fifth column represents the time duration of an eye fixation. The start and stop time values can be used to determine the time frame in which the eye fixations occurred.

The eye fixations during a time frame can be described as

(3) $$\begin{array}{c} E_{M_{T},T}=\left\{\;e_{m_{1},UR},e_{m_{2},UR\times 2},\ldots ,e_{m_{UR\times m\times 60},UR\times m\times 60}\;\right\}, \end{array}$$

where *E*_*M*_*T*_,*T*_ is the set of eye fixations that occurred for each time frame.

**Figure 7:**
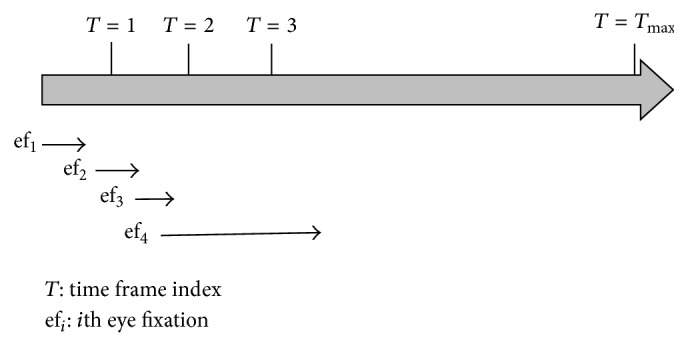
Example of eye fixation durations: the lengths of the arrows represent the eye fixation durations.

Figure 7 shows an example of eye fixation durations that occurred over the time frames. The time frames are based on the object movement update rate (i.e., objects would make discrete short burst of movements), and eye fixation durations can either fall within a time frame or stretch over more than one time frame.

**Step 2 (develop different types of AOIs).**

**Figure 8:**
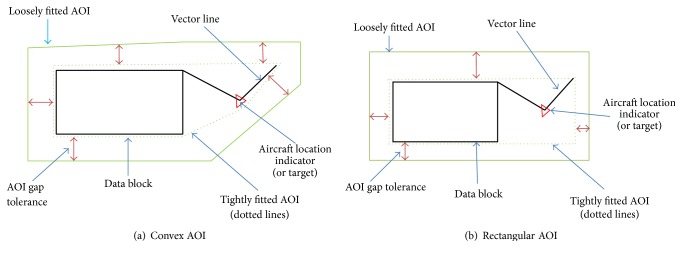
Example of a multielement object (black and red) represented using dynamic AOIs (green solid and dotted lines surrounding the object): the shape created when using dotted green lines represents a tightly fitted AOI, and the shape created when using solid green lines represents a slightly enlarged AOI with a buffer of 40 pixels.

Based on the preprocessed data from Step 1, different types of AOIs were developed. Two types of dynamic AOIs are considered: convex AOI and rectangular AOI. The rectangular AOI is an adaptation from [9], and in this research the shape and size of the rectangular AOI change based on each time frame. The convex AOI was developed by calculating the convex hull [19, 20] of the set of coordinate points used to represent each multielement object. The convex AOIs change their shapes and sizes based on each time frame as well.  Figure 8 represents the two different types of AOIs (convex and rectangular) for a multielement object. Thus, if an eye fixation occurs within a dynamic AOI, then we conclude that an eye fixation occurred on the multielement moving object.

To define a parameter that governs the size of the buffer, we define the buffer as the “AOI gap tolerance (AGT).” Since any given AOI corresponds to only one multielement object, *P*_*N*_*T*_,*T*_ can be substituted by AOI_*N*_*T*_,*T*_, the set of AOIs during a time frame, as

(4) AOINT,T=aoinUR,UR,aoinUR×2,UR×2,…,aoinUR×m×60,UR×m×60.

**Step 3 (map eye fixation data with AOIs).**

The “AOI mapping (AM)” performs a match between the eye fixation set and the AOI set during the same time frame. AOI mapping identifies whether the eye fixations fell within the boundaries of the AOIs by comparing the coordinates. The AM can be expressed as

(5) $$\begin{array}{c} AM:E_{M_{T},T}⟶AOI_{N_{T},T}. \end{array}$$

The functional mapping described in (5) is called a many-to-many mapping. Many-to-many mapping refers to the fact that eye fixations can be mapped to more than one AOI index and similarly AOIs can also be mapped to more than one eye fixation during a time frame. For example, in a single time frame, two or more eye fixations (that have different pixel-coordinates) can occur within a single AOI, or two or more AOIs can share a single eye fixation (when overlapping). The resulting mapped AOIs during the time frame *t* can be expressed as AM(*e*_*m*_*t*_,*t*_) = aoi_*n*_*t*_,*t*_. The collection of all mapped AOIs can be defined as a “mapped AOI set (MA)” and be written as

(6) $$\begin{array}{c} MA_{I,T}=\left\{\;{ma}_{i,t}\;|\;AM\left(\;e_{m_{t},t}\;\right)=aoi_{n_{t},t},{ma}_{i,t}\in ao{i_{n_{t}}}_{,t}\;\right\}, \end{array}$$

where MA_*I*,*T*_ is the set of mapped AOIs during a time frame and *I* is index.

**Figure 9:**
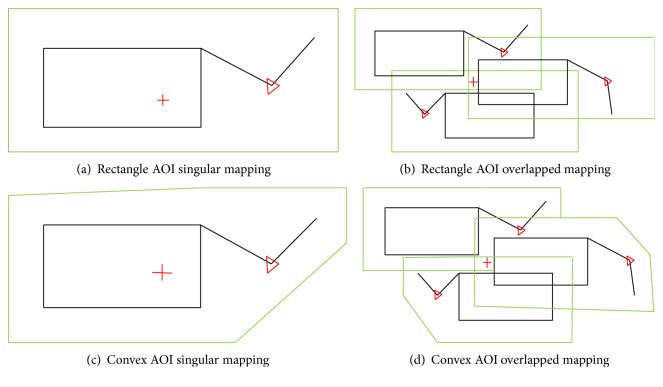
Mapping eye fixation with different AOI types: the red “+” indicates the eye fixation location. For (b) and (d), we determine that an eye fixation occurred in all three AOIs.

Figure 9 represents a mapping example where the rectangular and convex AOIs are shown in green. The red “+” symbol represents the eye fixation point that falls within the AOI boundary. There may be situations when an eye fixation falls inside the boundary of more than one AOI simultaneously. In other words, the eye fixation falls into a region that is in the intersection of several AOI boundaries, thus giving rise to the concept of “overlapped AOI mappings.” Thus, in this example, the mapped AOI set for this eye fixation will include three elements, which can be shown as MA_*I*,*T*_ = {*a*_1,*t*_, *a*_2,*t*_, *a*_3,*t*_}.

Another important concept, which will be useful in the analysis, is the cardinality of the MA set, where cardinality is the number of elements present in that set. This can be expressed as follows:

(7) $$\begin{array}{c} \left|\;ma_{i,t}\;\right|=c,\;c\in \left\{0,1,2,3,\ldots ,n_{t}\right\}, \end{array}$$

where |·| is the cardinality function and *n*_*t*_ is the number of multielement objects present at time *t*.

Thus, if “*c*” is the cardinality of the ma_*i*,*t*_ set we can say that the corresponding eye fixation index has been mapped to “*c*” number of AOIs simultaneously. The larger the cardinality of the ma_*i*,*t*_ set, the greater the difficulty in analyzing those eye fixations. Therefore, an important consideration in the data analysis is the frequency distribution of different cardinal values of the ma_*i*,*t*_ set.

**Step 4 (visualize plotted eye fixation data on the simulated scenarios).**

After the mapping process, the eye fixation data is overlaid on the simulated display as a function of time using the update rate. This process requires subsequently plotting both the eye fixations and AOI data pertaining to the same time frames and covering the time frames sequentially. Example cases are shown in Figure 12.

**Step 5 (investigate the mapping effects for different AOI gap tolerance (AGT) values).**

Some of the metrics that are of particular interest for this study are (1) the “percentage of the number of eye fixations falling inside AOIs (PNFIA)” and (2) the “percentage of the duration of the eye fixations falling inside AOIs (PDFIA).”

PNFIA is defined as

(8) PNFIA=Number  of  eye  fixations  falling  inside  AOIsTotal  number  of  eye  fixations,

where the number of eye fixations falling inside the AOIs (in (8)) is

(9) Number  of  eye  fixations  falling  inside  AOIs=∑t=1maxT ∑i=1mtAi,t,

where max⁡(*T*) is maximum value of the time frame count and *m*_*t*_ is the number of eye fixations during time frame *t*:

(10) $$\begin{array}{c} A_{i,t}=\left\{\begin{array}{cc} 1 & if\;\;\left|ma_{i,t}\right|\neq 0\\ 0 & otherwise, \end{array}\right. \end{array}$$

where the cardinality function is expressed as |·| (e.g., |ma_*i*,*t*_|).

*A*_*i*,*t*_ is the indicator function that becomes 1 if the cardinality of the corresponding set ma_*i*,*t*_ is nonzero; in other words this function takes the value of 1 if the associated eye fixation falls at least within one AOI boundary. Therefore, using (9) and (10) we get

(11) $$\begin{array}{c} PNFIA=\frac{\sum_{t=1}^{max\left(T\right)}\sum_{i=1}^{m_{t}}A_{i,t}}{N}, \end{array}$$

where *N* is the total number of eye fixations.

PDFIA is defined as

(12) PDFIA=Time  duration  of  eye  fixations  falling  within  AOIsTotal  time  duration  of  alleye  fixations.

The time duration of eye fixations falling within AOIs is calculated as

(13) $$\begin{array}{c} D=\overset{\max\left(T\right)}{\underset{t=1}{\sum }}\;\overset{m_{t}}{\underset{i=1}{\sum }}d_{i,t}, \end{array}$$

where *d*_*i*,*t*_ is time duration of eye fixation index *i* during time frame *t* and *m*_*t*_ is the number of eye fixations that occurred during time frame *t*.

For the purpose of calculating the time duration of eye fixations falling within an AOI, we need to consider only those eye fixations indexes for which the cardinality of their corresponding AOI mapped set is nonzero. Therefore we can use the indicator function described in (10) to take into account only those specific eye fixation indexes that fall at least within one AOI boundary. Thus we get the following:

(14) $$\begin{array}{c} D^{\prime }=\overset{\max\left(T\right)}{\underset{t=1}{\sum }}\;\overset{m_{t}}{\underset{i=1}{\sum }}A_{i,t}\times d_{i,t}. \end{array}$$

Using (13) and (14) we get that the percent time duration of eye fixations falling within an AOI to be

(15) $$\begin{array}{c} PDFIA=\frac{D^{\prime }}{D}. \end{array}$$

The next metric of interest is the frequency distribution of ma_*i*,*t*_ of various cardinalities. In other words, it is the frequency distribution of various possible “*c*” values, where *c* is as described in (7). This can be found by counting the number of occurrences of various possible values of “*c*.” This frequency distribution is an important metric because it is a qualitative measure of the difficulty associated with the analysis of the eye fixation sequence.

**Step 6 (change AOI gap tolerance (AGT) values).**

Due to the visual angle error, the choice of the AGT value depends on the discretion of the analyst. In absence of any established relationship between the AGT values and the relevant eye fixation parameters discussed in an earlier section, the optimal range of the AGT value becomes very much context dependent. As a result, it becomes important to study this relationship for the present context. Thus, the next step involves varying the AGT value to investigate its impact on the relevant metrics of interest. The equation governing the change in AGT can be written as

(16) $$\begin{array}{c} AGT_{R+1}=AGT_{R}+\delta , \end{array}$$

where AGT_*R*_ is AOI gap tolerance value for the iteration value *R* and *δ* represents increments of AGT values (e.g., *δ* = 5 pixels).

Table 2 shows the various values of the iteration counter *r* and the associated AGT values. All the above-mentioned steps need to be performed from Steps 2–5 for each *r* value.

**Step 7 (find optimal AOI gap tolerance value).**

Assuming that a participant or a group of participants interrogate one object at a time, one method to find the optimal AGT value is to select the AGT value that provides the highest frequency of the mapped AOI set of cardinality 1, or in other words we can identify the optimal AGT value for which the number of eye fixations on single AOIs is maximum.

The equation to find the optimal AGT value (AGT_optimal_) is as follows:

(17) $$\begin{array}{c} AGT_{optimal}=\underset{\mathrm{AGT}\in \left[5,10,\ldots ,100\right]}{\arg\;\max}\left[\;freq\left(\;c\;\right):c=1\;\right], \end{array}$$

where *c* is cardinality of the mapped AOI set and freq(·)  is frequency of set with cardinality value *c*.

Note that we can also obtain an overall single near optimal AGT value recommended for an experiment if we used the aggregated eye tracking data obtained from multiple participants.

Pseudocode 1 shows the simplified pseudocode based on the algorithmic flowchart shown in Figure 5.

## 3. Implementation

The developed approach was benchmarked through retired professional air traffic control specialists (ATCSs) who primarily work as instructors for the Federal Aviation Administration (FAA). The experiment was held at the FAA Civil Aerospace Medical Institute (CAMI), located in Oklahoma City, OK.

### 3.1. Participants

Ten certified ATCSs with over 32 years of experience participated in the experiment. In addition, three FAA employees participated as pseudo pilots who maneuvered the aircraft based on the controllers' clearances. Eye tracking data were collected from the certified controllers. Due to the unforeseen technical issues when using the eye tracking system and the air traffic control simulator, the data obtained from the first five participants were discarded, and only the data obtained from the subsequent five participants were used.

### 3.2. Apparatus

The experiment environment closely resembled the actual environment in the field (Air Route Traffic Control Center) in order to obtain high face validity. The simulated air traffic scenarios were displayed using a 19.83 × 19.83-inch monitor (2048 × 2048-pixel active display area). The size and resolution were equivalent to the actual display size used in the field. An additional monitor was placed to the right of the simulation monitor to display the En Route Automation Modernization (ERAM) tool, a decision support tool that provides text data with respect to aircraft data, trajectory, and possible conflicts. A keyboard was placed beneath the simulation monitor for an ATCS to input commands.

The eye tracking data were only collected from the simulation monitor to test our designs and algorithms. Facelab 5 eye tracker system [11] was used to collect the eye tracking data with a sampling rate of 60 Hz. The threshold for defining a fixation was set at 100 ms. The accuracy of the eye tracker was in the range of 0.5°–1° of visual angle error. Each participant's eyes were approximately in the range of 55–70 cm from the simulated display. Kongsberg-Gallium I-Sim software, internally outsourced and used by the FAA, was used for generating three different air traffic scenarios. The refresh rate of the simulated radar display was 1 second. Obtained raw eye tracking data was exported through the Eyeworks software [21], and the data output was similar to that shown in Table 1.

The structure of the air traffic simulation file is provided in Table 3 (sample data). The output file contains the details of the aircraft movements, their coordinates, and other relevant details of the aircraft representation used for the simulation. The data update rate (UR) was 1 second. In Table 3, the first and second columns show the elapsed time from the start of the experiment and the actual time of day, respectively. The third column named “aircraft code” shows the code name of the aircraft under consideration. The fourth column is the “target” column which shows the horizontal (*X* pos) and vertical (*Y* pos) coordinates of the targets (aircraft) in pixels. The fifth column is the “data block” column which has three subparts: (1) top left corner coordinates of the data block, (2) bottom right coordinates of the data block, and (3) direction column that represents the relative location of the aircraft with respect to the target position (N (north), NE (northeast), E (east), SE (southeast), S (south), SW (southwest), W (west), and NW (northwest)). The last column provides the position coordinates in pixels of the vector line's end point.

### 3.3. Task and Scenarios

**Figure 10:**
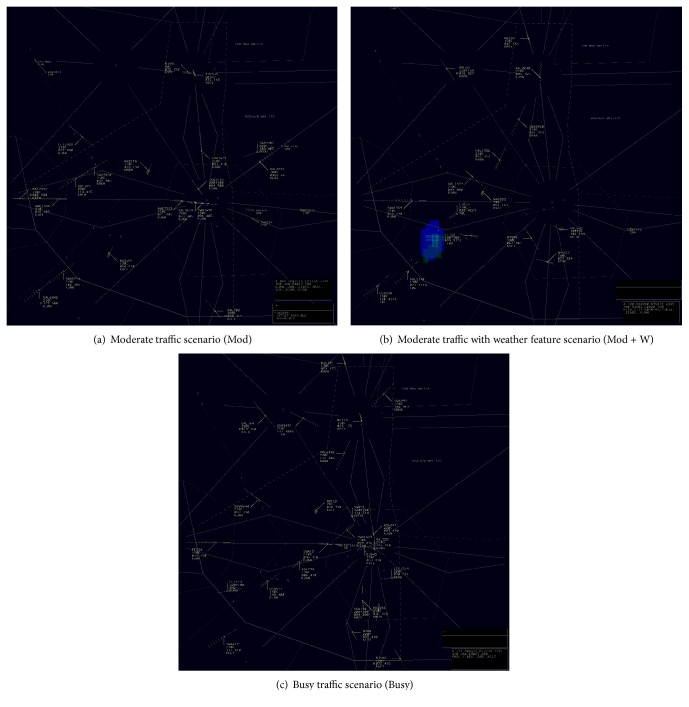
Air traffic control scenarios.

The task was a high fidelity representation of air traffic control as performed in the U.S. National Airspace System's Air Route Traffic Control Centers. Controlling simulated traffic such as this requires an experienced ATCS to observe the radar screen and give clearances to aircraft adjusting their altitudes, headings, or speeds so as to maintain aircraft-to-aircraft separation and route aircraft through the sector or to their destination airport within the sector. The ATCSs gave voice commands, via the communication system, to pseudo pilots who were situated in a remote room. The pseudo pilots followed the clearances and provided read-back to the ATCSs. Three scenarios were used (moderate traffic, moderate traffic with convective weather, and busy traffic). The duration of each scenario was 20 minutes.  Table 4 and Figure 10 show the details of the scenarios. In Figure 10(b), the blue patch represents the weather feature.

### 3.4. Data Analysis

The analysis of convex and rectangular AOIs was automated as follows: Based on the provided simulation output and the eye tracking output, both data sets were synchronized (step (1) in Figure 5). After the preprocessing steps, the two types of AOIs (convex and rectangular AOI) were created using the aircraft coordinates at every second (step (2)). Then, mapping was performed using the eye tracking data and the simulation data (step (3)). The mapped data was visualized (step (4)), and relevant metrics including the PNFIA and PDFIA were calculated by varying the AGT values (steps (5) and (6)). Finally, the optimal AGT value was obtained by identifying the highest percentage of the eye fixations on single AOIs (step (7)).

The complexity of the data processing time was *O*(*n*_1_ *n*_2_ *n*_3_ *n*_4_ *n*_5_ *n*_6_), where *n*_1_ is the number of participants, *n*_2_ is the number of scenarios, *n*_3_ is the number of AOI types, *n*_4_ is the number of AGT values, *n*_5_ is the number of AOIs per time frame, and *n*_6_ is the number of eye fixations per time frame. Each eye fixation was compared with each AOI per time frame.

In the Results, the total eye fixation numbers and durations on the display (without using AOIs) were plotted in order to investigate the oculomotor trends. Then, aggregated PNFIA and PDFIA values for all participants were plotted based on the AGT values. Then, the number of eye fixations that occurred on single and multiple overlapping AOIs was plotted based on the AGT values. The optimal AGT value was computed, and examples of different scanpath sequences (resulting from either different AOI types or AGT values) were identified.

## 4. Results

**Figure 11:**
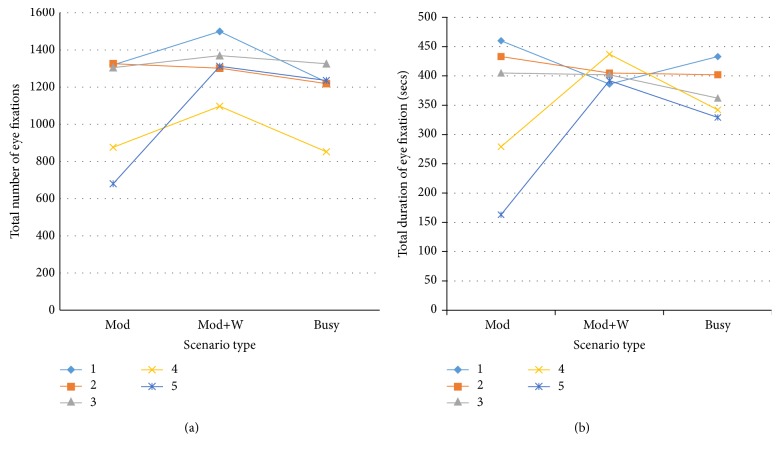
Oculomotor trends of the total number and duration of eye fixations among scenarios.

The oculomotor trends are shown in Figure 11.  Figure 11(a) shows the total number of eye fixations and Figure 11(b) shows the total duration of eye fixations with respect to scenario difficulties: moderate traffic (Mod), moderate traffic with weather feature (Mod + W), and busy traffic (Busy). The legends in Figure 11 showing 1, 2, 3, 4, and 5 represent the participant numbers.

**Figure 12:**
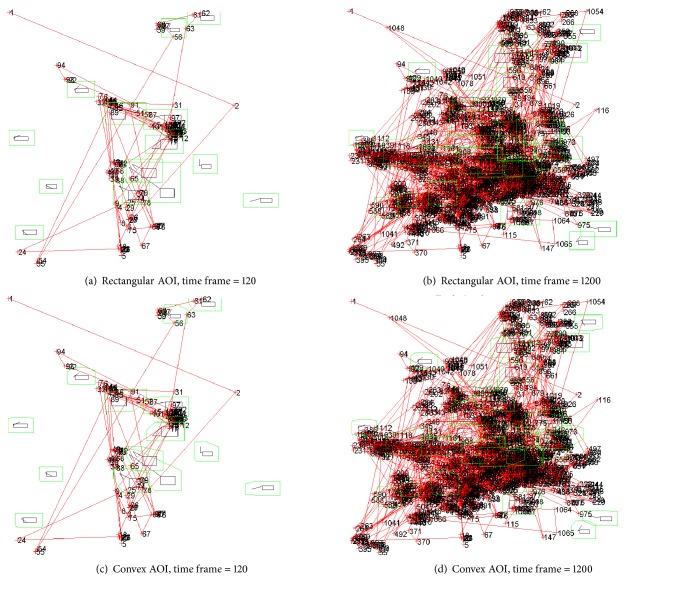
Examples of visual representations of eye fixation data plotted onto the AOIs: the eye movements (eye fixation orders are numbered, and the saccadic movements are shown as red lines) were accumulated over time.

Figure 12 displays example snapshots of the visualization process (see Step (4) in Figure 5) for both AOI types. The example snapshots show the dynamic AOIs with the AGT value set to 40 pixels. In Figure 12, the AOIs are highlighted in green and the order of eye fixations along with the associated saccades (connections between eye fixations when moving from one to the next) are highlighted in red. Note that the automated illustrations of the ordered eye fixations (shown in numbers) and the saccades linking the eye fixations are accumulated, meaning that the illustrations show all eye fixations from the scenario start time (time frame 1) until the indicated time frame such as time frame 120 or 1200.

**Figure 13:**
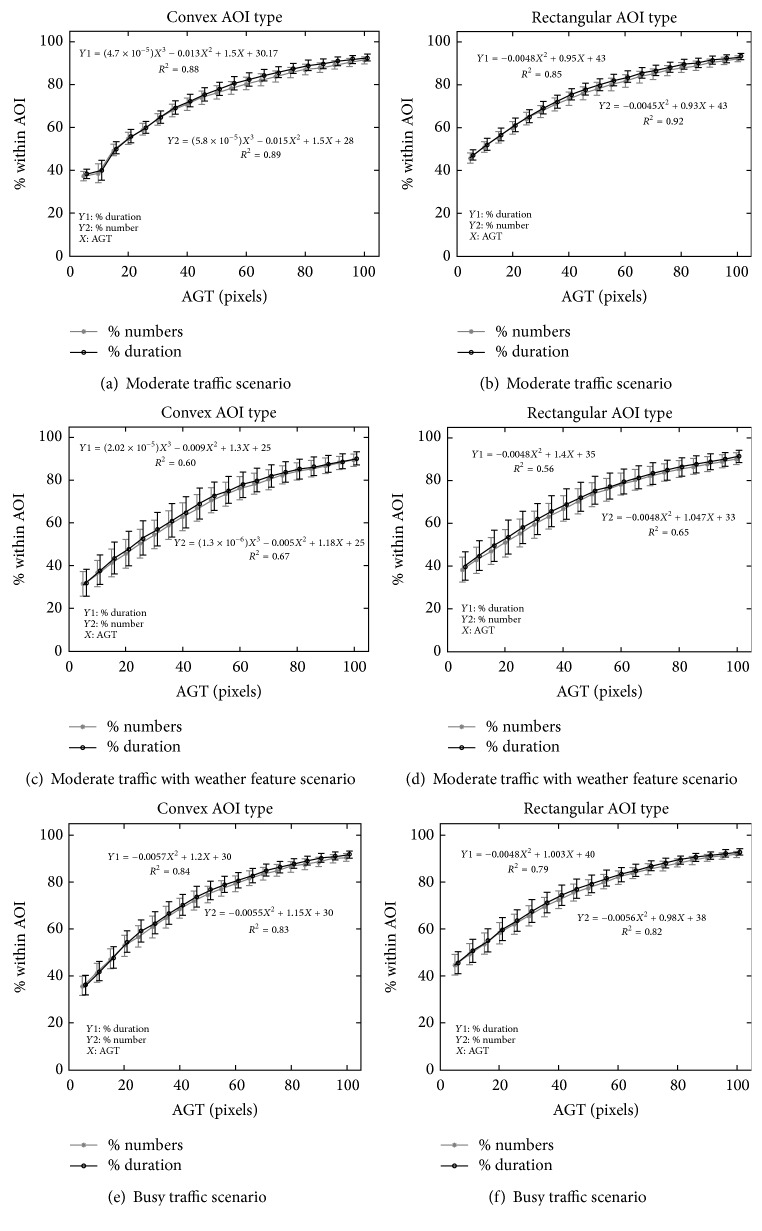
Plots of coverage percentages of the numbers and durations of the eye fixations that occurred within the AOI versus AGT values: the figures on the left column are the results for the convex type, and the figures on the right column are the results for the rectangular type.

Figure 13 depicts the effect of changing the AGT values on (1) the percentages of the numbers of eye fixations that fall within AOIs (PNFIA) shown in grey and (2) the percentages of the durations of the eye fixations that fall within AOIs (PDFIA) shown in black. The plots show the mean and standard error associated with every AGT value. In addition, the fitted polynomial equations and the *R* ^2^ values are provided.

**Figure 14:**
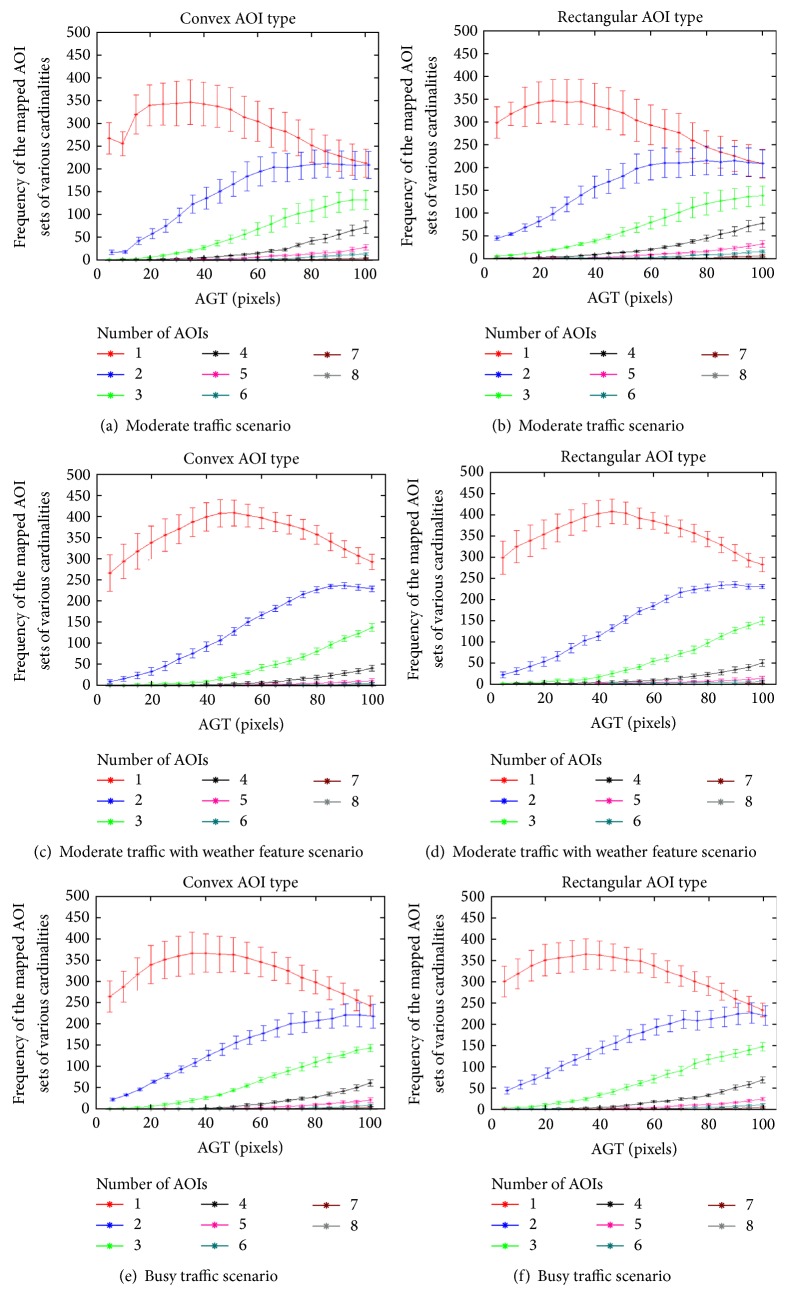
Distribution of the number of eye fixations on single or overlapped AOIs based on AGT values: the top red line shows the change of the number of eye fixations for a single AOI. The subsequent lines show the change of the number of eye fixations on overlapping AOIs (increasing from 2 to 8).

Figure 14 depicts the change in the frequency of mapped AOI sets, of various cardinalities, with respect to the change in AGT values for convex and rectangular AOIs, respectively. The plots show the mean and the standard error associated with the coverage percent values. The maximum possible observed cardinality of the mapped AOI set is 8. A general trend among the various plots is that the frequency count of the ma_*i*,*t*_ set having cardinality 1 (or in other words *c* = 1 (shown in red)) increased and then decreased. As the AGT values increased, the number of overlapping AOIs also increased, and the eye fixations on a single AOI subsequently decreased.

The near optimal (or recommended) AGT values (by considering all participants and scenarios) are provided in Table 5. The AGT value of 40 pixels captures approximately 70–80% of the total eye fixations that fall within the AOIs. Note that the participants can freely observe other areas that are not defined as AOIs within the display.

**Figure 15:**
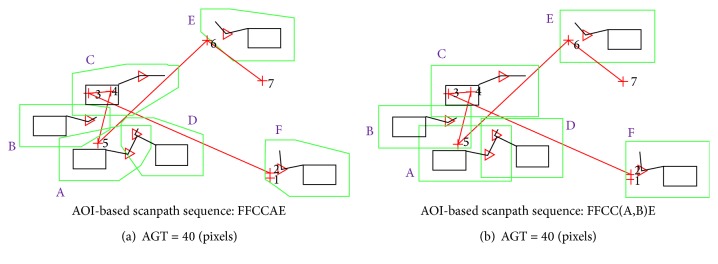
Examples illustrating how AOI types can affect the AOI-based scanpath sequences: the red “+” shows the location of the eye fixations, and the numbers are the corresponding eye fixation orders. For (a), eye fixation 5 only falls inside AOI “B,” whereas for (b) eye fixation 5 falls inside both AOIs “A” and “B.”

Figure 15 depicts the change in the frequency of mapped AOI sets, of various cardinalities, with respect to the change in AGT values for convex and rectangular AOIs, respectively. The plots show the mean and standard error associated with the frequency values for every AGT value. The maximum possible observed cardinality of the mapped AOI set is 8. In many cases the frequency of cardinality values higher than five was zero. Thus the curves for these cardinalities might not be exclusively visible on the plots as they are overlapping each other. A general trend among the various plots is that the frequency count of the ma_i,t_ set having cardinality 1 (or in other words *c* = 1 (shown in red)) increased and then decreased. As the AGT values increased, the number of overlapping AOIs also increased, and as a result, the eye fixations on a single AOI subsequently decreased.

**Figure 16:**
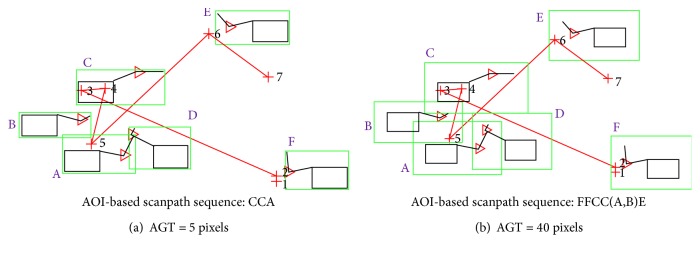
Examples illustrating how AGT values can affect the AOI-based scanpath sequences: the red “+” shows the location of the eye fixations, and the numbers are the corresponding eye fixation orders. For (a), eye fixations 1, 2, 6, and 7 fall outside the AOIs, whereas for (b) only eye fixation 7 falls outside the AOIs.

Figure 16 shows examples of how different AGT values can affect the resulting AOI-based scanpath sequences. More relevant eye fixations were captured when using the optimal AGT value of 40 (obtained from our experiment) than the AGT value of 5. As shown in Figure 16, the identified scanpath sequence “FFCC(A,B)E” (Figure 16(b)) shows much more pertinent mappings compared to the scanpath sequence “CCA” (Figure 16(a)). Again, note that the scanpath sequences can be further collapsed into “FC(A,B)E” and “CA,” respectively.

## 5. Discussion

An approach was developed that automatically (1) created rectangular and convex AOIs around multielement objects, (2) mapped eye fixations with different types of AOIs, (3) systematically evaluated the mapping characteristics by increasing the size of the AOIs to consider the fidelity of the eye trackers, and (4) investigated how the increase of the AOI sizes affects the overlapping of multiple AOIs. This approach was applied to the collection of visual scanning data from a high fidelity simulation of an air traffic control task. The task required ATCSs to interrogate multielement moving objects (that can change their overall shapes) on a radar display. The approach was applied to eye tracking data collected from the ATCSs as they performed the conflict detection and control task through interrogating multiple moving aircraft within their sector.

The oculomotor statistics on different types of scenarios show that the overall eye fixation numbers and durations on the display (without considering AOIs) did not significantly differ among the scenarios. The results differ from previous aircraft conflict detection research [22, 23]. In [22], eye fixation numbers and durations increased as the difficulty level increased (easy: many aircraft had different altitudes; moderate: many aircraft had similar altitudes; difficult: many aircraft changed altitudes), while setting the number of aircraft on the display at twelve for all scenarios. In [23], eye fixation numbers and durations increased as the number of aircraft on the display was increased from twelve to twenty. A major difference in the scenario settings was that there was no time limit on detecting possible collisions for [22, 23], whereas the experiment in this research had a time limit of twenty minutes.

Regarding the ATCSs' cognitive processes, one reason that similar oculomotor trends could be found is that the ATCSs were constantly vigilant on interrogating and controlling the aircraft throughout the experiment. In addition, the reason for a marginal decreasing trend on eye fixations and durations may be due to the order effect of the scenarios being performed in a sequence of moderate traffic, moderate traffic with convective weather, and busy traffic. The participants could have become more comfortable with the situation as they continued to control the multiple aircraft. Another possibility is that the ATCSs may have spent more time on looking at the ERAM display as well as the keyboard. Unfortunately, the exported eye tracking data only provides pixel-based eye fixations that occurred within the defined display; therefore, it is difficult to know where the eye fixations occurred outside the display.

The convex and rectangular AOI types did not generally affect the amount of mapped eye fixations among the participants and the scenarios due to the relatively small size of the objects as well as the accuracy of the eye tracking system for a high face validity experiment. However, we were able to identify specific examples of different AOI types affecting the resulting scanpath sequence (Figure 15). The analysis of human performance using the scanpath sequences may have substantially differed for the same experiment if the analysts applied different AOI types. The effect may have been overall significant if the size of the multielement objects was bigger due to the increased unnecessary area (Figure 8) created by the rectangular AOI type. The unnecessary areas would also result in creating more overlapping AOI areas.

The AGT values substantially impacted the amount of covered eye fixations and durations on both AOI types and the trends fitted to polynomial equations. Up to a certain point, the increase of the AGT value was able to accommodate many eye fixations that occurred around the objects; then the increase rate (of the amount of included eye fixations) began to reduce since lower amount of eye fixations occurred further away from the objects. The eye fixation numbers and durations were highly correlated for our experiment. Note that the AGT values also affected the resulting scanpath sequences (Figure 16). The use of too tightly fitted AOIs resulted in missing many eye fixations that occurred around the object. Note that if we used AOIs that were too large, then the cardinality of the mapped AOI set would increase, leading to either inaccurate mapping or an increase in the complexity of the scanpath sequences by having more overlapping AOIs.

Thus, the selection of the AGT value gives rise to a trade-off between the coverage (amount of eye fixations) versus complexity (overlapping AOIs) of the algorithm because the more we increase the coverage, the more we increase the complexity. As the AGT value increases, the coverage of the overlapping AOIs increases accordingly, but the coverage of the single AOIs starts to decrease (Figure 14). The reason is that overlapping AOIs begin to take away the amount of eye fixations that occurred within single AOIs. Therefore, we were able to determine the near optimal AGT value by identifying the coverage peak of single AOIs. Having an adequate AOI size to map an eye fixation to a single AOI is more preferred to having larger AOIs that would result in creating unnecessary overlapping areas. In other words, the more we increase the coverage, the more we increase the complexity for multielement moving objects that can overlap.

## 6. Limitation and Future Research

Although the different AOI types did not show significant differences when aggregated results were compared, we were able to identify specific cases where differences were indeed present. A follow-up experiment is needed to vary the size of the actual objects in order to identify a threshold that shows substantial mapping differences when using complex convex approximations versus the simple shaped approximations. In addition, although the benchmarking of the developed methods was able to show that trade-offs exist when considering the design of AOIs based on visual angle errors and overlapping objects, more follow-up experiments are needed to refine and better support our methods.

In addition, the near optimal AGT values were obtained from aggregated data across the whole experiment and among the participants. The limitation to this approach is that we apply a constant AGT value for the whole duration. The optimal AGT value might not be a constant for all the time frames, and further detailed analysis might help to segregate time segments from the whole experimental duration (i.e., identify the amount of variations for different segregated time segments). Note that we would not be able to obtain a trend to identify the optimal value if the time length was too short (e.g., for a 1-second time frame, we would only obtain 1 or 2 eye fixations). To investigate how it would vary, we would first need to define the time segments that we should apply.

Another limitation is that we assumed that the multielement objects make discretized movements and that the scene (background) is fixed. If the background is moving or the objects make rapid movements (e.g., from one end of the screen to another end of the screen in a very short time), then our approach would not work. These issues are difficult to solve and should be addressed in our subsequent research.

The overarching goal of our research is to obtain more accurate mappings between the eye movements and the moving objects in order to better support the analysis of human performance. This research concentrated on prototyping, implementing, and evaluating new conceptual designs and algorithms to obtain more accurate mappings. Based on the obtained results in this research, we are currently analyzing the human performance based on the obtained AOI-based scanpath sequences through the Directed Weighted Networks [24, 25].

Furthermore, the results can be a basis to develop better scanpath analysis methods that build upon existing methods [22, 26–31], mimic human performance [32], and develop data visualization methods for active learning using the experts' visual scanning patterns [33]. In addition, the visual scanning data could be combined with EEG analysis [34] to better understand how the different types of tasks or incidents affect brain response and visual scanning and how the brain response data is correlated with visual scanning data.

## 7. Conclusion

To address the issue of mapping eye fixations with multielement objects (that move, can change their shape, and overlap over time), we proposed and implemented dynamic AOIs that represent the multielement objects. During the process, we showed a way to map eye fixations to overlapping AOIs. In addition, the concept of AGT was applied in order to address the issue of the fidelity of the eye trackers. Our approach was automated and applied to data collection from a high fidelity simulation of an air traffic control task. The benchmark showed that eye tracking data analyses can substantially differ based on how the AOIs are defined and how we can obtain near optimal values to better define the AOIs.

## Figures and Tables

**Pseudocode 1:**
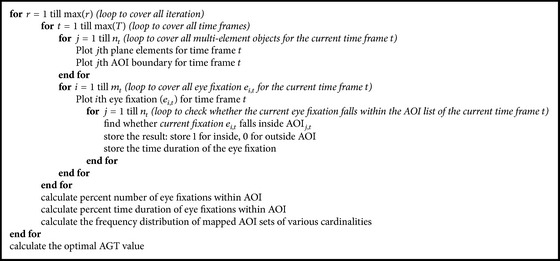
Pseudocode used for the overall process.

**Table 1:** Example of eye fixation data.

*X* pos (pixels)	*Y* pos (pixels)	Start time (secs)	Stop time (secs)	Duration (secs)
1047	1668	0.35	0.466	0.116
628	1255	0.816	1.15	0.334
852	1174	1.5	2.233	0.733
1150	1690	2.666	2.916	0.25
1162	1721	2.933	3.416	0.483

**Table 2:** AGT values defined for each iteration (*r*).

Various combination of *r* and AGT values	
*r*	AGT (pixels)
1	5
2	10
3	15
4	20
5	25
6	30
7	35
8	40
9	45
10	50
11	55
12	60
13	65
14	70
15	75
16	80
17	85
18	90
19	95
20	100

**Table 3:** Air traffic simulation sample output data.

Scenario time	Time of the day	Aircraft code	Target		Data block					Vector line end point	
					Top left		Bottom right		Direction		
			*X* pos	*Y* pos	*X* pos	*Y* pos	*X* pos	*Y* pos		*X* pos	*Y* pos
00:00:14	11:55:19	DAL1268	988	939	1050	922	1141	994	E	957	892
00:00:14	11:55:19	EGF1819	599	1248	608	1282	699	1354	S	624	1237
00:00:14	11:55:19	N21LD	732	1300	792	1334	883	1406	SE	742	1277
00:00:15	11:55:20	DAL1268	987	938	1050	922	1141	994	E	957	892
00:00:15	11:55:20	EGF1819	599	1248	446	1231	537	1303	W	624	1237
00:00:15	11:55:20	N21LD	732	1299	792	1334	883	1406	SE	742	1277

**Table 4:** Characteristics of different simulation scenarios.

Scenario name	Average unique aircraft per frame	Min unique aircraft per frame	Max unique aircraft per frame	Std dev unique aircraft per frame
Moderate traffic	20	7	30	7
Moderate traffic + weather feature	20	6	29	6
Busy traffic	24	8	37	7

**Table 5:** Mean and standard error for the optimal AGT values for different AOI types.

Optimal AGT	AOI type	
	Convex AOI	Rectangular AOI
Mean (pixels)	40	38.3
Standard error (pixels)	1.8	1.2
